# Polygenic predictors of age-related decline in cognitive ability

**DOI:** 10.1038/s41380-019-0372-x

**Published:** 2019-02-13

**Authors:** Stuart J. Ritchie, W. David Hill, Riccardo E. Marioni, Gail Davies, Saskia P. Hagenaars, Sarah E. Harris, Simon R. Cox, Adele M. Taylor, Janie Corley, Alison Pattie, Paul Redmond, John M. Starr, Ian J. Deary

**Affiliations:** 1 Centre for Cognitive Ageing and Cognitive Epidemiology, The University of Edinburgh, Edinburgh, UK; 2 Department of Psychology, The University of Edinburgh, Edinburgh, UK; 3 Social, Genetic and Developmental Psychiatry Centre, King’s College London, London, UK; 4 Centre for Genomic and Experimental Medicine, Institute of Genetics and Molecular Medicine, The University of Edinburgh, Edinburgh, UK; 5 Alzheimer Scotland Dementia Research Centre, The University of Edinburgh, Edinburgh, UK

**Keywords:** Psychology, Predictive markers

## Abstract

Polygenic scores can be used to distil the knowledge gained in genome-wide association studies for prediction of health, lifestyle, and psychological factors in independent samples. In this preregistered study, we used fourteen polygenic scores to predict variation in cognitive ability level at age 70, and cognitive change from age 70 to age 79, in the longitudinal Lothian Birth Cohort 1936 study. The polygenic scores were created for phenotypes that have been suggested as risk or protective factors for cognitive ageing. Cognitive abilities within older age were indexed using a latent general factor estimated from thirteen varied cognitive tests taken at four waves, each three years apart (initial *n* = 1091 age 70; final *n* = 550 age 79). The general factor indexed over two-thirds of the variance in longitudinal cognitive change. We ran additional analyses using an age-11 intelligence test to index cognitive change from age 11 to age 70. Several polygenic scores were associated with the level of cognitive ability at age-70 baseline (range of standardized *β*-values = –0.178 to 0.302), and the polygenic score for education was associated with cognitive change from childhood to age 70 (standardized *β* = 0.100). No polygenic scores were statistically significantly associated with variation in cognitive change between ages 70 and 79, and effect sizes were small. However, *APOE* e4 status made a significant prediction of the rate of cognitive decline from age 70 to 79 (standardized *β* = –0.319 for carriers vs. non-carriers). The results suggest that the predictive validity for cognitive ageing of polygenic scores derived from genome-wide association study summary statistics is not yet on a par with *APOE* e4, a better-established predictor.

## Introduction

Mean levels of several cognitive functions decline as people grow older, even in those without dementia. This is observed for many important cognitive abilities, such as memory, processing speed, and reasoning [1–3], with so-called “crystallized” abilities, such as vocabulary, less affected. There is strong evidence that age-related declines across all abilities are correlated: cognitive ageing, as with individual differences in cognitive ability level, is substantially a general phenomenon [4–6]. Declines in cognitive abilities in older age have practical consequences for daily life and independent living: they have been linked to lower ability to perform everyday functions such as understanding medicine labels [7], and to increased vulnerability to financial fraud [8, 9]. Discovering predictors of variation in cognitive ageing might help us to identify those at highest risk of more rapid decline, and—to the extent that such predictors are confirmed to be causal—devise appropriate interventions. In the present study, we assessed the value of a panel of genetic risk scores in predicting variation in general cognitive decline in a generally healthy sample across the eighth decade of life.

Many studies have investigated whether variables that are known to correlate cross-sectionally with cognitive ability are also predictive of variation in its decline. Numerous such factors have been tested, but few have been replicated consistently [10]. For instance, although higher educational attainment has been found in some studies to predict shallower rates of cognitive decline [11, 12]—a finding which has informed theories of “cognitive reserve” [13]—other studies have not found this same effect [14, 15]. Other potential predictors, with varying degrees of evidentiary support, and a great deal of methodological heterogeneity, include physical fitness, as measured by variables such as grip strength and lung function [16, 17] (see ref. [18] for a review), personality traits such as conscientiousness [19] (see ref. [20] for a review), and type 2 diabetes [21] (see ref. [22] for a review).

Here, we investigate potential genetic predictors of cognitive level at age 70, and relative cognitive decline from age 11 to 70 years, and from age 70 to 79 years. One such predictor is well-known already: carriers of either one or two *APOE* e4 alleles (as opposed to no such alleles) are not just at higher risk of a diagnosis of Alzheimer’s disease [23, 24], but also appear to be at risk of steeper cognitive decline [25]. In recent years, however, a new method has become commonly used in research investigating genetic prediction of traits: the calculation of polygenic scores. This method uses summary data from published genome-wide association studies (GWAS) that have tested the correlations of millions of single-nucleotide polymorphisms (SNPs) with phenotypes of interest. Using the weightings (regression coefficients) for each SNP from these data, genotyped individuals in an independent sample (one not included in the original GWAS) can have a polygenic score (PGS) calculated that indexes the cumulative small effects that produce a genetic liability to a certain disease, or a probability of a higher level of a particular trait [26]. Through meta-analysis, and through the collection of ever-larger datasets, the sample size, and thus the statistical power, of GWAS studies continues to increase. For example, the variance explained in educational attainment in independent samples by the educational attainment PGS has increased alongside the sample size of the discovery GWASs [27, 28].

PGSs can be used to predict variables other than their “own” phenotype. The PGS for educational attainment, for example, has been shown not just to predict educational attainment but also, among other variables, cognitive ability [29], the rate of cognitive development in childhood [30], social mobility [30, 31], and longevity [32]. It is possible, then, that the genetic variants linked to phenotypic predictors of the cognitive ability or relative cognitive decline may also themselves predict later-life decline [33]. Indeed, a number of papers have found links between various disease-linked polygenic scores and older age cognitive ability, including some indications at earlier data-collection waves of the same sample examined here [34–37].

Testing PGSs as predictors of outcomes such as cognitive level and change is potentially useful and efficient. Researchers or clinicians can use a single source material—a participant’s DNA—to test their genetic propensity to a very wide range of risk and protective factors [38]. Therefore, instead of having to measure all the phenotypes that might confer risk to or protection of cognitive decline, it might be possible—to the extent that those phenotypes are heritable and have had a large, high-quality GWAS performed—to assess the genetic propensity to the phenotype and use that information to predict cognitive level and decline. The approach using PGSs, if successful, would also allow the retrospective testing of risk and protective factors in cohorts where DNA and longitudinal cognitive data are available but who were never tested for the risk or protective factors in question. Beyond these potential strengths, PGSs can even be used to assess propensity to a phenotype that is never expressed, such as liability to schizophrenia in a sample in which no one develops the illness [26]. Finally, understanding the genetic variants that are and are not shared among different traits and disorders may provide clues to their biological aetiologies.

We selected fourteen PGSs based on, first, the relevant phenotype having been linked to cognitive decline in at least one previous study and, second, on there being a recent GWAS of that phenotype (Table S1 provides a list of references to the phenotypic studies, and to the respective GWASs). The PGSs in question were those for the following variables: educational attainment, the personality traits of neuroticism and conscientiousness, Alzheimer’s disease, Parkinson’s disease, schizophrenia, major depressive disorder, coronary artery disease, stroke, type 2 diabetes, smoking, height, body mass index, lung function, and grip strength. We tested the associations of each of these PGSs with the level (at age 70 years) and age-related slope (from age 70 to age 79 years) of general cognitive ability estimated from a battery of thirteen varied tests. We added a further analysis where we tested the association of the PGSs with the change between a cognitive test taken at age 11 and age-70 general cognitive ability. We tested their predictive value individually, simultaneously, and—because the presence of the *APOE* e4 allele has previously been found to predict cognitive decline in this same cohort during almost the same period of life [5]—in models also including the *APOE* e4 status of the participants.

## Methods

### Sample

The Lothian Birth Cohort 1936 (LBC1936) is an ongoing longitudinal study of older, community-dwelling individuals living mostly in the Edinburgh and Lothians area of Scotland, UK [39, 40]. They were recruited on the basis of their having been part of the Scottish Mental Survey of 1947 [41], and have, to date, attended four testing waves: the first at mean age 69.54 years (SD = 0.83; *n* = 1091; 543 females), the second at age 72.52 years (SD = 0.71; *n* = 866; 418 females), the third at age 76.25 years (SD = 0.68; *n* = 697; 337 females), and the fourth at age 79.32 (SD = 0.62; *n* = 550; 275 females). For simplicity, we will henceforth refer to the ages at each wave as 70, 73, 76, and 79 years, respectively. Ethical approval for the LBC1936 study came from the Multi-Centre Research Ethics Committee for Scotland (MREC/01/0/56; 07/MRE00/58) and the Lothian Research Ethics Committee (LREC/2003/2/29). All participants, who were volunteers and received no financial or other reward, completed a written consent form before any testing took place.

### Cognitive measures

In addition to completing the Moray House Test No. 12 at age 11 years [42], which measures a variety of cognitive domains with an emphasis on verbal reasoning, the LBC1936 members completed a wide selection of cognitive tests at each of the later-life testing waves. Tests were administered identically at each occasion. Thirteen tests were used for the present analysis, covering the four broad cognitive domains described below. Participants also completed the Mini-Mental State Examination (MMSE) [43] at each wave; results from wave 1 were used to validate the Alzheimer’s disease PGS (see below).

*Visuospatial ability* was measured using tests of pattern-based reasoning, recognition, and recall: the Matrix Reasoning and Block Design subtests of the Wechsler Adult Intelligence Scale, 3rd UK Edition (WAIS-III^UK^ [44]), and the Spatial Span subtest of the Wechsler Memory Scale, 3rd UK Edition (WMS-III^UK^ [45]; the score used here was an average of forwards and backwards spatial span).

*Verbal memory* was measured using three tests of recall of new verbal information: the Logical Memory and Verbal Paired Associates subtests of the WMS-III^UK^ (both indicated by their total score), and the Digit Span backwards subtest of the WAIS-III^UK^.

*Crystallized ability* was measured by three tests: the National Adult Reading Test (NART [46]), the Wechsler Test of Adult Reading (WTAR [47]) and a test of phonemic verbal fluency [48]. All three tests assessed prior verbal knowledge.

*Processing speed* was measured using four tests tapping cognitive speed in a variety of ways. Two of the tests were pencil-and-paper “clerical” tasks: the Digit-Symbol Substitution and Symbol Search tasks from the WAIS-III^UK^. A third was a psychophysical measure of Inspection Time performed on a computer monitor (as described in ref. [49]). A fourth was a test of Choice Reaction Time, measured using the dedicated button-box described in ref. [50]. Note that, in each of the analyses, we reversed scores on the Choice Reaction Time test so that higher scores indicated better (faster) cognitive performance.

### Genetic measures

The majority of participants provided blood samples at the age 70 wave that were used to extract DNA for the genetic analyses. To measure single-nucleotide polymorphisms (SNPs) we used the Illumina 610-Quadv1 whole-genome SNP array; measurements were completed at the Wellcome Trust Clinical Research Facility Genetics Core, Western General Hospital, Edinburgh. Polygenic scores (PGSs) were created using PRSice software [51], with linkage-disequilibrium clumping parameters set to *r*^2^ > 0.25 over 250 kb sliding windows. All PGSs were calculated using all SNPs from their respective GWAS (see Table S1 for all references and discovery sample sizes); that is, we used an association threshold of *p* ≤ 1.00. In three cases (smoking, lung function, and grip strength), we ran a new GWAS on data we had available from the UK Biobank sample (see Supplementary Method and Figures S1–S3). This was either because this resulted in a larger GWAS than the most recent published GWAS at the time, or because the LBC1936 participants were included in that most recent GWAS. In addition to the PGS analyses, each participant’s *APOE* e4 genotype was ascertained using TaqMan technology, also at the Wellcome Trust Clinical Research Facility Genetics Core. Since there were few carriers of two *APOE* e4 alleles (~2% of the sample), we categorised this variable as the binary presence (306 participants; ~30%) or absence (722 participants; ~70%) of any *APOE* e4 alleles.

### Statistical analysis and preregistration

In a set of preliminary analyses, we estimated whether each polygenic score was significantly associated with its “own” phenotype in the Lothian Birth Cohort. We selected phenotypes that were as closely-related as possible given the data we had available. The selected phenotypes were as follows. For the education PGS, we used years of education, reported at age 70. For the Neuroticism and Conscientiousness PGSs, Neuroticism and Conscientiousness were estimated using the NEO-FFI personality instrument [52], self-reported at age 70. For the Alzheimer’s disease we used the scores on the MMSE, and for the Schizophrenia PGS, we used WAIS-III Block Design (since this test provided an estimate of cognitive ability, which is impaired in schizophrenia; no test of schizophrenia symptoms was available). For the major depressive disorder PGS, we used the score on the depression subscale of the Hospital Anxiety and Depression Scale (HADS [53]), taken at age 70. For the coronary artery disease, stroke, and type 2 diabetes PGSs, we used self-reports of whether the participants had ever received a diagnosis of any of these conditions by age 70. For the smoking PGS, we used a self-report of whether the participant was a never-, ex-, or current smoker at age 70. For the height and BMI PGSs, we used the measurements of these traits taken by nurses at the age-70 testing wave. Finally, for the FEV_1_ and grip strength PGSs, we used the measurements of these physical functions taken at age 70 using a spirometer and a dynamometer, respectively.

The analyses described below were preregistered, except for the final one (including age-11 intelligence scores to estimate lifetime cognitive change), which, therefore, should be considered as exploratory. The time-stamped preregistration document, written after data from the fourth testing wave of LBC1936 were collected and entered into the database, but before any of these data had been seen by any of the analysts of this study, can be found at the following URL: https://osf.io/vyy4u/.

Before analysing any of the cognitive data, we used a parallel analysis, using the *psych* package for R [54], to factor-analyse the PGSs, testing whether there was evidence for a general factor (as there is for cognitive tests). If evidence of such a general factor emerged, we planned to assess the value of this general factor as a predictor in the cognitive models.

**Fig. 1:**
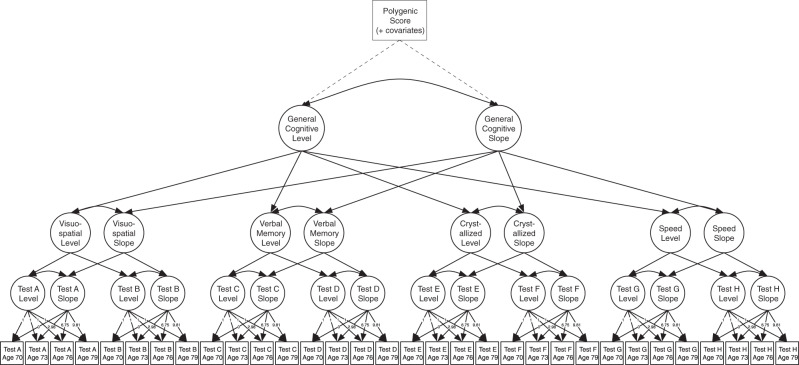
Simplified diagram of the structural equation model used to estimate the general cognitive level and the general cognitive slope. A latent growth curve was estimated across the four waves for each cognitive test (with the numbers showing the average length between each wave), and the levels and slopes were factor-analysed in a hierarchical model with four cognitive domains and a general factor. Note that, for illustrative purposes, not all tests are shown (the Speed domain had four tests and the other domains had three each; see Table 2). The main outcomes of interest—the association of each polygenic score with the general level and slope variables—are indicated by the dashed lines

We estimated a “factors of curves” structural equation model to characterise cognitive levels and changes within older age. This involved estimating a latent growth curve model for each cognitive test, then factor-analysing the latent intercepts and latent slopes from these models. The model follows the same structure as that of ref. [5], where we used data from the first three waves of the LBC1936 to examine predictors of cognitive change from age 70 to 76 years. The factor models for both levels and slopes were hierarchical, as shown in Fig. 1: there were four domain-level factors estimated for both level and slope (Visuospatial ability, Verbal Memory, Crystallized ability, and Processing Speed), which were themselves factor-analysed to produce the general factors of cognitive level and slope. Squaring the product of each test’s loading on its domain and the domain’s loading on the general factor provided a proportion of variance in each test explained by the general factor; averaging these values produced the mean amount of variance explained at each level: the general factor level, the domain level, and the test level.

To estimate the mean change in each cognitive ability over time, we first ran a model with the raw cognitive scores. The models where we included the genetic predictors had the cognitive scores pre-residualised for age in days at the time of testing and for sex. Where possible, the analyses were run within the structural equation model (that is, the factors of curves model and the association with the predictors were estimated simultaneously). However, in some cases where the structural equation model would not converge, we extracted factor scores for the general factors and used linear regression to predict them from the genetic predictors. All such models are noted below.

We next ran the further analyses testing each PGS as a predictor alongside (that is, controlling for) *APOE* e4 status, to test for incremental predictive validity over this well-established risk factor. We did this only for PGSs that had shown a significant relation to the relevant cognitive outcome in the main analysis. Then, we included all the PGSs together as predictors in a single model, assessing their incremental validity over one another. For a final analysis, which was not part of the preregistration, we tested whether each genetic predictor (the PGSs and *APOE* e4) was associated with age-70 general cognitive ability after correcting for the age-11 intelligence measure. In this way, we were able to test whether each measure was predictive of cognitive change across most of the life course (that is, between ages 11 and 70).

The LBC1936 participants had a homogeneous, White European background, and thus we did not expect population stratification to have a strong influence on the results. Nevertheless, for all analyses involving relating the PGSs (and *APOE* e4) to phenotypes, we included four SNP principal components (multidimensional scaling components) as covariates. We used the False Discovery Rate correction [55] to adjust *p*-values for multiple comparisons across the 15 predictors (14 PGSs plus *APOE* e4). Structural equation modelling was performed using Mplus v7.3 [56], and used full-information maximum likelihood estimation (FIML) to use all of the available data in each model. Foster et al. [57] recommend that the missing-data technique is chosen on the basis of its assumptions being plausibly fit to a dataset; here we operated under the “missing at random” assumption—the basis for FIML—where patterns of missingness were assumed not to be systematically related to the missing cognitive scores (see discussion in ref. [5]). All other analyses were run in R [58].

## Results

### Preliminary polygenic score analyses

Eleven of the fourteen PGSs were significantly associated with their “own” related phenotype (see Table 1) in the LBC1936 sample. These significant relations ranged from explaining 1.00% of the variance, in the case of the grip strength PGS in predicting grip strength, to the PGS for height explaining 13.07% of the variance in height. Three of the scores were not significantly related to the outcome phenotype: the PGS for Alzheimer’s disease was not significantly correlated with the dementia-screening Mini-Mental State Examination score (explaining 0.38% of the variance, *p* = .051), the PGS for major depressive disorder was not related to HADS depression score (explaining less than .001% of the variance, *p* = 0.94), and the PGS for stroke was not significantly related to self-reported stroke (explaining 0.42% of the variance, *p* = 0.24).

**Table 1:** Variance explained by each polygenic profile score in relevant Lothian Birth Cohort 1936 outcome variables

Polygenic profile score	Outcome variable	Std. *β*	SE	*p*-value	% Variance explained
Educational attainment	Years of education	**0.276**	**0.031**	**1.55** × **10**^**−19**^	**7.58%**
Neuroticism	NEO-PI-R Neuroticism	**0.176**	**0.038**	**1.85** × **10**^**−06**^	**2.58%**
Conscientiousness	NEO-PI-R Conscientiousness	**0.083**	**0.033**	**0.014**	**0.69%**
Alzheimer’s disease	MMSE	–0.061	0.031	0.051	0.38%
Schizophrenia	Block design	**–0.120**	**0.034**	**4.57** × **10**^**−04**^	**1.23%**
Major depressive disorder	HADS depression score	0.002	0.032	0.941	5.49 × 10^−04^%
Coronary artery disease	Cardiovascular disease^†^ (24.5%)	**0.267**	**0.076**	**3.94** × **10**^**−04**^	**1.89%**
Stroke	Stroke^†^ (5.0%)	0.169	0.145	0.243	0.42%
Type 2 diabetes	Type 2 diabetes^†^ (8.6%)	**0.608**	**0.122**	**7.27** × **10**^**−07**^	**5.79%**
Smoking	Smoking	**0.147**	**0.032**	**5.41** × **10**^**−06**^	**2.05%**
Height	Height	**0.365**	**0.030**	**1.72** × **10**^**−34**^	**13.07%**
BMI	BMI	**0.359**	**0.031**	**5.78** × **10**^**−31**^	**11.85%**
FEV_1_	FEV_1_	**0.191**	**0.026**	**3.00** × **10**^**−13**^	**5.21%**
Grip strength	Grip strength	**0.065**	**0.020**	**0.002**	**1.00%**

Standardized *β*s, SEs, and *p*-values are from general linear (continuous outcomes) or generalized linear (categorical outcomes, indicated with the † symbol and with the percentage of the sample who reported having, or having had, that condition at or by age 70 in parentheses) regression models adjusting for age at the time of measuring/reporting the outcome variable, sex, and four multidimensional scaling components. All significant *p*-values remained significant after False Discovery Rate correction. For continuous outcome variables, the % variance explained is derived from the partial *R*^2^. For categorical outcome variables, the % variance explained is derived from the Nagelkerke’s *R*^2^. Rows in bold survived false discovery rate correction for multiple testing. References for the GWAS source of each polygenic score can be found in Table S1

*NEO-PI-R* NEO-Personality Inventory-Revised, *MMSE* Mini-Mental State Examination, *HADS*  Hospital Anxiety and Depression Scale, *FEV1*  Forced expiratory volume in 1 second

A correlation matrix of the relations among each of the PGSs is provided in Table S1. The PGS for education showed the highest number of significant relations to the other PGSs, being related to nine of the other scores; this mostly consisted of education-linked variants being positively associated with variants linked to better health (broadly consistent with evidence from genetic correlations; see e.g., ref. [59]). The correlation sizes among the PGSs were generally low: the strongest absolute relation between any of the PGSs was that between the education and BMI PGSs, estimated at *r* = –0.26. Despite its poor phenotypic prediction of the depressive state as described above, the PGS for major depressive disorder correlated in the expected direction with that for neuroticism (*r* = 0.23) [60, 61]. As planned in the preregistration, we ran a parallel analysis of the fourteen PGSs using the *psych* package for R: this indicated that there were four factors in the data, with no evidence for a strong “general” factor. Therefore, we did not use any of these factors in the analyses below.

### Cognitive levels and slopes

**Fig. 2:**
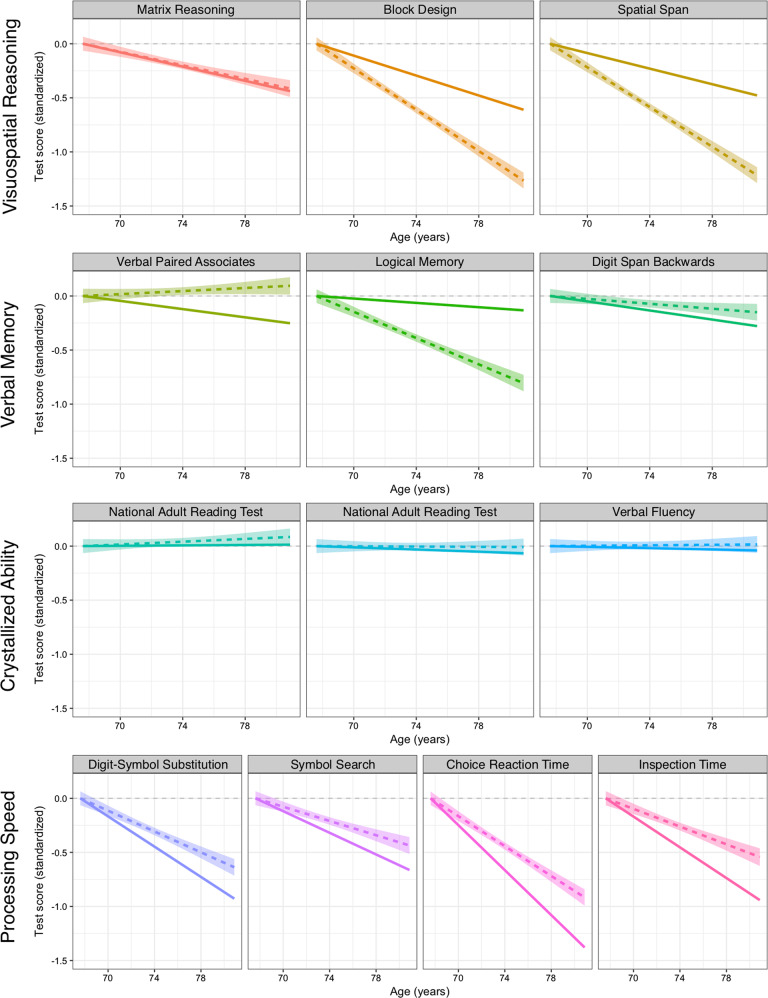
Standardized linear trajectories of each cognitive test with age. Intercepts (at the youngest age) are set to zero for comparative purposes. The horizontal dotted line indicates zero. The coloured solid line is the model-implied trajectory (using full-information maximum likelihood estimation); the coloured dotted line is the regression line through the raw data (with shaded 95% confidence interval)

Descriptive statistics for each of the cognitive tests at each wave, and a full correlation matrix, are provided in Tables S3 and S4 in the Supplementary Materials, respectively. The longitudinal changes, estimated from the structural equation models, for each cognitive test score are shown in Table 2. All but two of the tests showed statistically significant declines over time, with the largest per year declines being seen in the processing speed tests. The two tests showing no significant age-related change were the NART and verbal fluency, both of which were in the category of “crystallized” tests and were thus expected to show less decline with age. The trajectory of each test with age is illustrated in Fig. 2, for the model-implied trajectory as well as the change in the raw data for comparison (see Figure S4 for an alternative way of visualizing the data, showing each data point). In all but one case the model-implied trajectory was the same in sign as that in the raw data, though some slopes were different in magnitude. For verbal paired associates, the magnitude was reversed (it became negative in the model). These changes are to be expected given the use of FIML estimation in the structural equation model: several previous methodological investigations have shown that the choice of missing-data technique can influence results [54, 55].

**Table 2:** Estimates of the linear slope of each cognitive test. Illustrations of each trajectory are shown in Fig. 2

Domain	Cognitive test (max. score)	Mean (SD) at age-70 baseline	Mean raw change per year	SE of raw change	*p*-value	Mean no. of SDs change per year
Visuospatial ability	Matrix reasoning	13.49 (5.13)	–0.133	0.016	6.12 × 10^−16^	–0.033
	Block design	33.79 (10.32)	–0.423	0.029	1.30 × 10^−49^	–0.046
	Spatial span	7.36 (1.42)	–0.038	0.005	1.27 × 10^−15^	–0.036
Verbal memory	Logical memory	71.46 (17.96)	–0.150	0.072	.038	–0.010
	Verbal paired associates	26.44 (9.13)	–0.156	0.033	3.00 × 10^−06^	–0.019
	Digit span backwards	7.73 (2.26)	–0.038	0.007	1.72 × 10^−07^	–0.021
Crystallized ability	NART	34.48 (8.15)	0.012	0.013	0.379	0.001
	WTAR	41.02 (7.17)	–0.034	0.012	0.003	–0.005
	Verbal fluency	42.42 (12.54)	–0.032	0.035	0.358	–0.003
Processing speed	Digit-symbol substitution	56.60 (12.93)	–0.833	0.038	5.44 × 10^−105^	–0.070
	Symbol search	24.71 (6.39)	–0.258	0.023	1.50 × 10^−28^	–0.050
	Inspection time	112.14 (11.00)	–0.595	0.049	2.26 × 10^−34^	–0.071
	Choice reaction time (ms)	64.21 (0.09)	–0.008	3.57 × 10^−04^	1.86 × 10^−108^	–0.104

Estimates of SD change are model-implied, using full-information maximum likelihood estimation, and thus may not precisely correspond to the raw SDs

We then factor-analysed the levels and slopes of all the tests, as described above and shown in Fig. 1. The baseline model (with the tests corrected for age within the wave and sex, but with no polygenic score predictors) showed excellent fit to the data, according to multiple fit indices: *χ*^2^(1298) = 2446.16, *p* < 0.001; root mean square error of approximation = 0.028; comparative fit index = 0.968; Tucker-Lewis index = 0.967; standardized root mean square residual = 0.057. The full parameters for this model are shown in Figure S5 in the Supplementary Materials. The general factor of cognitive level explained 40.9% of the variance in performance across all thirteen cognitive tests’ scores, and, for the same tests, the general factor of cognitive slope explained 69.7% of the variance in cognitive change between ages 70 and 79.

### Polygenic score prediction of general cognitive level and slope

**Fig. 3:**
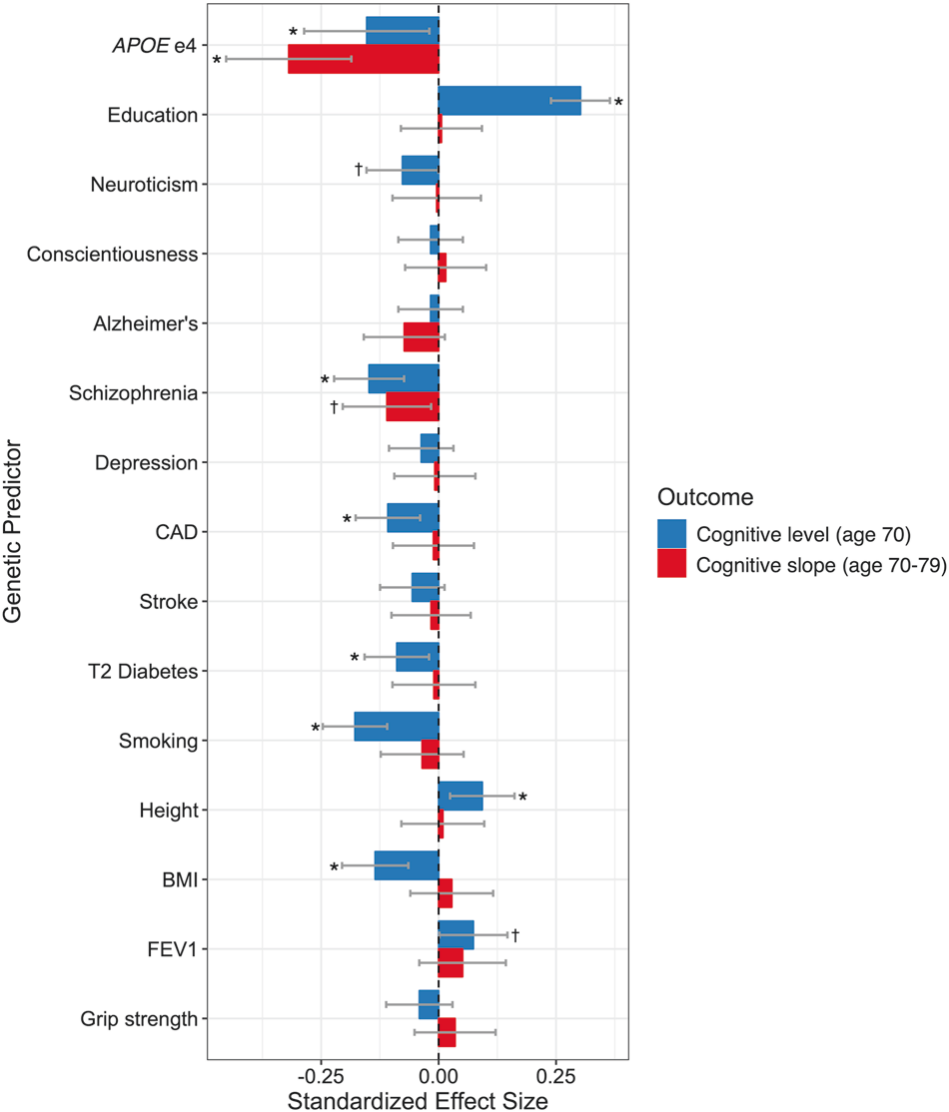
Associations of *APOE* e4 status and each polygenic score with cognitive level (age 70) and cognitive decline (age 70–79). * = Statistically significant after false-discovery rate correction. † = Nominally significant, but no longer significant after false-discovery rate correction. Note that the effect for *APOE* e4 is standardized only with respect to the outcome (with a dichotomous predictor); all other effect sizes are standardized with respect to both the outcome and the predictor

The associations of each PGS and *APOE* e4 with the age-70 level and age 70-to-79 slope of general cognitive ability are shown in Table 3 and Fig. 3. Of the fourteen PGSs, seven were statistically significantly associated with cognitive level at age 70: higher PGSs for education and height were associated with higher general cognitive levels, and PGSs for schizophrenia, coronary artery disease, type 2 diabetes, smoking, and BMI were associated with lower general cognitive levels. All effect sizes for these significant effects were modest: the standardized betas ranged from –.178 (for smoking) to .302 (for education); the *p*-values that remained significant after multiple-comparisons correction ranged from 1.31 × 10^−20^ to 0.008. *APOE* e4 status was also significantly associated with level: its association became non-significant after correction for multiple comparisons.

**Table 3:** Associations of each polygenic profile score, and *APOE* e4 status, with general cognitive level (age 70) and slope (age 70–79) in individual-predictor models

Genetic variable	Association with baseline *g*			Association with *g* slope		
	Std. *β*	SE	*p*-value	Std. *β*	SE	*p*-value
*APOE* e4	**–0.153**	**0.068**	**0.025**	**–0.319**	**0.068**	**3.44** × **10**^−**06**^
Education	**0.302**	**0.032**	**1.31** × **10**^−**20**^	0.006	0.044	0.888
Neuroticism	–0.077	0.039	0.047	–0.004	0.048	0.929
Conscientiousness	–0.017	0.035	0.634	0.015	0.044	0.726
Alzheimer’s disease	–0.017	0.035	0.634	–0.073	0.044	0.094
Schizophrenia	**–0.148**	**0.038**	**1.07** × **10**^**-04**^	–0.110	0.048	0.022
Major depressive disorder	–0.037	0.035	0.290	–0.008	0.044	0.856
Coronary artery disease	**–0.108**	**0.035**	**0.002**	–0.011	0.044	0.808
Stroke	–0.056	0.035	0.109	–0.016	0.043	0.719
Type 2 diabetes	**–0.089**	**0.035**	**0.012**	–0.010	0.045	0.826
Smoking	**–0.178**	**0.035**	**3.66** × **10**^**-07**^	–0.035	0.045	0.438
Height	**0.093**	**0.035**	**0.008**	0.009	0.045	0.833
BMI	**–0.135**	**0.036**	**1.76** × **10**^**-07**^	0.028	0.045	0.540
FEV_1_	0.074	0.037	0.048	0.051	0.047	0.277
Grip strength	–0.041	0.036	0.255	0.035	0.044	0.425

All estimates come from hierarchical latent growth curve structural equation models. Associations are corrected for age at cognitive testing, sex, and four multidimensional scaling components. Bold values are those that were statistically significant after false discovery rate correction for multiple testing. Note that the results for *APOE* e4 were estimated using extracted factor scores in linear regression models; all other effect sizes were estimated within the structural equation models themselves

Only one of the PGSs, that for schizophrenia, was a significant (negative) predictor of general cognitive slope between ages 70 and 79, but this did not survive multiple-comparisons correction. *APOE* e4 status, however, was a significant predictor of general cognitive decline—those with one or two *APOE* e4 alleles had significantly steeper general cognitive decline than those who had none—and this association survived multiple-testing correction.

We next tested whether the scores that had shown significant relations to the cognitive outcomes in the initial analysis were still statistically significantly related after including *APOE* e4 as a separate predictor. Results are shown in Table S5. In all cases, the polygenic score predictor remained significant after adjusting for *APOE* e4 status, with slightly attenuated effect sizes: that is, their associations seemed to be largely independent of any relation with *APOE* e4.

Next we ran the simultaneous-predictor models, including all PGSs predicting general cognitive level, and (in a separate model) general cognitive slope. For general cognitive level at age 70, only the significant associations with the PGSs for education (*β* = 0.217) and schizophrenia (*β* = –0.109) remained (see Table S6 for full results). As in the initial analysis, none of the PGSs were significantly related to general cognitive slope over time after correction for multiple comparisons.

Note that, due to the appearance of newer (and larger) GWAS studies during the submission and review process of this paper, we have updated several of the PGSs. The original results are provided in Table S11 (for the list of GWAS studies used for the main results in the paper, see Table S1).

### Additional (non-preregistered) analyses I: Polygenic score prediction of lifetime cognitive change

We ran the analyses predicting general cognitive level at age 70 adjusted for age-11 cognitive ability—that is, predicting cognitive change across most of the life course. The basic cognitive model is shown in Figure S6. Note that age 11 cognitive ability was related very strongly to age 70 general cognitive level (*β* = 0.814, SE = 0.015, *p* = ~0.00; see ref. [5]). Results from the genetic analyses are shown in Table 4, along with correlations between each of the genetic predictors and age-11 cognitive ability itself. Seven of the fourteen PGSs were significantly associated with intelligence score at age 11 after multiple-comparisons correction: education, schizophrenia, coronary artery disease, stroke, type 2 diabetes, smoking, and BMI. There was no association with *APOE* e4. The association between lifetime cognitive change and the PGS for education was significant (and survived multiple-testing correction), with a standardized effect size of *β* = .100: those with a higher PGS for education saw relatively less cognitive change across the lifespan. The other PGSs were not related significantly to the lifetime cognitive change variable.

**Table 4:** Associations of each polygenic score and *APOE* e4 status with age 11 intelligence and lifetime cognitive change (age 70 general intelligence (*g*) adjusted for age 11 intelligence)

Genetic variable	Association with age 11 intelligence			Association with age 70 *g*, adjusted for age 11 intelligence		
	Std. *β*	SE	*p*-value	Std. *β*	SE	*p*-value
*APOE* e4	.024	.074	.740	–.080	.055	.141
Education	**.321**	**.032**	**2.11** **×** **10**^**-24**^	**.100**	**.026**	**1.01** **×** **10**^**-04**^
Neuroticism	–.032	.037	.377	–.016	.027	.563
Conscientiousness	.010	.033	.741	–.171	.119	.153
Alzheimer’s disease	.012	.033	.692	–.033	.025	.174
Schizophrenia	**–.136**	**.036**	**1.85** **×** **10**^**-04**^	–.021	.028	.446
Major depressive disorder	.028	.033	.389	–.032	.025	.191
Coronary artery disease	**–.098**	**.032**	**.003**	–.039	.025	.116
Stroke	**–.075**	**.032**	**.022**	.016	.025	.523
Type 2 diabetes	**–.079**	**.033**	**.019**	–.010	.025	.703
Smoking	**–.187**	**.033**	**2.60** **×** **10**^**-08**^	–.023	.026	.369
Height	.063	.033	.056	.013	.025	.595
BMI	**–.164**	**.034**	**2.15** **×** **10**^**-06**^	.001	.079	.989
FEV_1_	.055	.035	.113	.023	.026	.380
Grip strength	–.030	.033	.362	–.002	.025	.927

*Note:* Lifetime change come from a hierarchical latent model of general cognitive ability, comprising tests taken at age 70 (Figure S3), with the *g*-factor adjusted for age 11 intelligence. Rows in bold are effects that survived (per-column) false discovery rate correction for multiple testing. All models included four principal components to adjust for population stratification

In addition, we ran a follow-up analysis to further probe this latter result (that the education PGS was related to cognitive change across the life course). Since there is evidence that cognitive abilities are improved by time spent in education [62], it is of interest to include phenotypic years of education in the analysis, and test whether the association between the education PGS and cognitive change is mediated by achieved years of education. We did so in a structural equation model where the education PGS had both a direct path to general cognitive ability at age 70, and an indirect one via phenotypic years of education (see Figure S7). As above, general cognitive ability at age 70 was regressed on the Moray House Test score at age 11, to form a life course cognitive change measure. We found a significant, but small, mediation effect such that 6.4% of the educational polygenic score’s relation to cognitive change was mediated by years of education. This is consistent with two conclusions: first, some of the genetic effect on cognitive change across the life course is independent of achieved years of education: there was still a residual direct path in the model. Second, the link between education and cognitive change is not fully a genetic effect; there is still the possibility that purely environmental effects of education are influencing cognitive change. We are grateful to an anonymous reviewer for suggesting this analysis; full results from the mediation model are in Table S7.

### Additional (non-preregistered) analyses II: further investigation of the Alzheimer’s disease score

We did not expect that the Alzheimer’s disease PGS would make such a poor prediction of general cognitive slope, especially when *APOE* e4 status had made a substantial prediction, and SNPs associated with *APOE* e4 featured prominently in Alzheimer’s GWAS results. We tested whether this was due to our use of the *p* = 1.00 threshold when calculating the PGS; it is possible that inclusion of the large number of less- and non-significant SNPs overwhelmed the signal from *APOE* e4-linked loci. Therefore, we recalculated the Alzheimer’s disease PGS at a more stringent threshold—*p* = 0.01—and re-ran the relevant analyses. As would be expected, the more stringent PGS had a somewhat higher (point-biserial) correlation with *APOE* e4 status (*r*(964) = .286, *p* = 4.82 × 10^−21^) than did the PGS with the *p* = 1.00 threshold (*r*(963) = 0.117, *p* = 2.73 × 10^−04^). The more stringent Alzheimer’s PGS also made a significant prediction of the slope of general cognitive decline (*β* = –0.109, SE = 0.043, *p* = 0.012), though not its initial level (*β* = –0.056, SE = 0.035, *p* = 0.110).

For comparison, we also recalculated the other PGSs at the *p* = 0.01 threshold. The results for all *p* = 0.01 PGSs are shown in Table S8. Most results were similar: unlike the Alzheimer’s result reported above, all other non-significant associations with general cognitive slope remained non-significant with this new threshold. The predictions for cognitive level generally had similar effect sizes, though the association between level and coronary artery disease was no longer significant, whereas the nominally-significant associations for the height PGS did not survive multiple comparisons correction.

### Additional (non-preregistered) analyses III: comparing genotypic and phenotypic prediction

A reviewer of the manuscript suggested that we compare the polygenic score predictions of cognitive ageing to the predictions made from their associated phenotypes. To this end, we took the phenotypes that were related to the polygenic scores above (under “preliminary polygenic score analyses”) and used them to predict general cognitive level and slope. The results, and comparisons using Williams’s test, are shown in Table S9. For level, some polygenic scores made near-identical predictions as their associated phenotype: this was the case for coronary artery disease, type 2 diabetes, smoking, height, and BMI. For education, neuroticism, and Alzheimer’s disease, the phenotype was a substantially better predictor than the polygenic score. For slope, all effect sizes were small, and the only significant phenotypic predictor after correction was that of the MMSE score (used as the Alzheimer’s-related phenotype); higher MMSE scores predicted slightly healthier cognitive ageing over time.

### Additional (non-preregistered) analyses IV: competing mortality risks across polygenic scores

Given that many of the LBC1936 members died during the progress of the longitudinal study, it is of interest to consider whether the competing risk of mortality that is potentially conferred by differences in some of the polygenic scores [32] might explain the null associations found above. To this end, as an additional analysis, we used the suite of polygenic scores, and *APOE* e4 status, to predict mortality (for which data were collected via the National Health Service’s Central Register, provided by the National Records of Scotland and provided to the LBC1936 team on approximately a 12-week basis). In the sample of 1,005 participants where polygenic scores were available, there had been 277 deaths (~25% of the starting sample) by the most recent update (April 2017). The average age at death was 77.00 years (SD = 3.37). The results are shown in Table S10: of the scores, only one—that for higher BMI—was significantly associated with a greater risk of earlier death, and this association was not significant after false discovery rate correction. In addition, we tested whether individuals who went on to die before the end of the follow-up period had a lower general intelligence at age 70, and a steeper slope of cognitive decline, but neither of these reached statistical significance (level: *β* = –0.062, SE = 0.035, *p* = 0.078; slope: *β* = –0.045, SE = 0.027, *p* = 0.095). Overall, then, there was little evidence of mortality-specific dropout being a strong biasing factor in the cognitive function-based results presented here.

## Discussion

The study reported here was an attempt to shed light on the genetic links between cognitive ageing and a range of traits and disorders by performing polygenic score prediction of age-related cognitive decline. All the PGSs were chosen *a priori* to index genetic variants associated with key ageing-relevant traits and conditions. Some of these PGSs were associated with the baseline cognitive level at age 70, and the education PGS was associated with cognitive change from age 11 to age 70. However, all of the PGSs had small, non-significant relations with the gradient of the general cognitive slope (based on a general cognitive decline variable that explained over two-thirds of the slope variance across all thirteen tests) between age 70 and 79. The presence of the *APOE* e4 allele, on the other hand, made more substantial predictions of cognitive decline: having either one or two alleles, compared to zero, explained around 10% of the variance in the slope of cognitive decline between age 70 and 79. None of the PGSs approached this level of effect size. Below, we discuss some of the implications, strengths, and limitations of the study.

Overall, the results were similar to results from studies attempting to use phenotypic data to predict cognitive decline (e.g. attempts in this same sample over a shorter period of change [5]): cognitive levels could be predicted with far larger effect sizes than cognitive slopes. It should be noted that some of the PGS correlations with cognitive level were similar or larger in effect size to those using the actual phenotypes themselves (see Table S9). For example, phenotypic BMI had a correlation of –0.130 with cognitive level at 70; the equivalent for the BMI PGS was –0.135. This similarity in the phenotypic and genotypic effect sizes was also the case for coronary artery disease, height, smoking, and type 2 diabetes. This was not, however, the case for predicting slope, where few if any variables showed substantial predictive effect sizes, and effect sizes were similarly small for both genotypic and phenotypic predictors.

It may be that any genetic effects indexed by the PGS occur at different points in the life course. For instance, variants linked to education and BMI—two PGSs that were significantly associated with baseline cognitive level, but not slope—may have their effects on cognitive ability during childhood or early adulthood, whereas any effects of *APOE* e4—which significantly predicted slope, but not level—may appear only within older age. In one GWAS of general cognitive ability [63], the effect of *APOE* e4 was particularly pronounced at older ages, consistent with these findings (see ref. [64] for a longitudinal analysis in an even older sample, and ref. [65] for an investigation of age-dependent pleiotropy among cognitive traits and psychiatric disorders). Note that any PGS associations reported herein were independent of *APOE* e4, as per our simultaneous regression analysis including them both as predictors.

The PGSs that were associated at with cognitive ability level at age 70 were generally the same as those that showed associations at age 11 (that is, those for education, schizophrenia, coronary artery disease, stroke, type 2 diabetes, smoking, and BMI); however, only the PGS for education showed an association with later ability after correcting for early ability—that is, only the PGS for education was correlated with lifetime cognitive change. This result implies that education-linked variants are related to changes in cognitive abilities before older age (specifically, before age 70). These findings are of relevance to the concept of “cognitive reserve” [13]: they imply that researchers who find links between early-life education and later-life cognitive abilities (or cognitive change) should take into account the fact that some of the effect may come from a genetic propensity to better educational attainment, and not the educational attainment itself.

It may simply take a higher-powered study, with more participants, longer follow-up times, and additional waves, to detect genetic effects with the small sizes of those suggested here. On the other hand, as was noted above, larger GWAS studies have tended to produce PGSs with better predictive validity [27], and this will probably also be the case for future iterations of the PGSs studied here. Another reason for the small-sized predictions may be because the phenotypes linked to the PGSs do not themselves reliably predict cognitive decline. The most recent systematic review [10] noted that much of the evidence in this sphere is weak; we do not have a strong, multi-study evidence base for many of the phenotypic—let alone genetic—predictors of cognitive decline discussed here. It is also possible that genetic propensities interact with environments in ways that improve or worsen the cognitive ageing trajectory. Note that there is no existing large, well-powered GWAS of cognitive decline in particular, since there are too few samples with the relevant variables for such a GWAS to be run. Should such a study appear in future, we would expect to derive a PGS that would predict cognitive decline in independent samples.

The case of the Alzheimer’s PGS warrants further consideration. As noted above, we chose to calculate all PGSs at the most liberal, whole-genome threshold (*p* = 1.00), including the effects of all SNPs, rather than a more restricted set. The choice of just one single threshold was to avoid the overfitting that often comes with choosing many thresholds and reporting the one with the highest association with the outcome trait of interest. However, the fact that the prediction of cognitive decline by *APOE* e4 status was so much larger than that of the Alzheimer’s PGS, which itself contains the effect of (SNPs in linkage disequilibrium with) *APOE* e4, suggested that the effects of the other SNPs—less significantly or not significantly related to cognitive decline—included in the PGS were overpowering the *APOE* e4-linked signal. This appeared to the be the case: the use of a more conservative threshold improved the predictive validity. This was not the case for the education PGS, however: it may be that, for traits that have even higher levels of polygenicity—for which there are no large-effect variants such as *APOE* e4 for Alzheimer’s—the PGS threshold matters less. As we noted above, larger GWASs of Alzheimer’s disease will produce summary data with more signals from non-*APOE*-e4-linked variants, and these should be tested for their association with normal-range cognitive ability and cognitive decline at different thresholds; we note that a previous study [66] found no relation between a PGS calculated from an older Alzheimer’s GWAS and cognitive abilities in this same sample (see also ref. [67] for an example of a combination of *APOE* and PGS variables). Alternatively, performing a permutation test as described in ref. [51] (Supplementary Note 4) would allow the calculation of an empirical *p*-value threshold, potentially allowing for a better prediction than we had with our across-the-board use of the *p* = 1.00 threshold. Generally, however, there are as yet no hard-and-fast rules for the use of PGSs by researchers who wish to maximise their predictive ability but are concerned about multiple-comparisons testing; ref. [68] provides some recommendations.

Another strategy to improve prediction would be to follow the approach of ref. [69], where the authors took a much larger set of eighty-one PGSs and used them as predictors of the levels of various traits including cognitive ability and BMI (they used a sample of younger individuals and thus could not examine cognitive decline). They ran their analysis using penalized regression (specifically the Elastic Net [70]), which allowed the large number of PGS variables to be reduced to the best set of predictors, and allowed them to explain a somewhat larger proportion of the variance in the traits. Such an hypothesis-free method, using algorithmic selection from many predictors, rather than manually choosing those predictors on the basis of pre-existing links to cognitive decline, as we did here, may be a more fruitful approach for prediction (if not theoretical understanding). Finally, given the evidence that risk for particular conditions increases substantially at the very high end of some polygenic score distributions [71], it may be possible in a larger sample than the one used in the present study to isolate individuals with extreme polygenic scores related to, for example, specific disorders, and investigate whether they show pronounced rates of cognitive decline.

### Strengths and limitations

The LBC1936 sample is a rare dataset in that it is a narrow-age sample (all participants were from a single year of birth) in which the follow-up waves cover nearly a decade within older age, with thirteen high-quality cognitive tests measured repeatedly and identically; moreover, and highly-unusually for an ageing study, it has well-validated cognitive test data from age 11. Using longitudinal structural equation modelling, we estimated general factors of cognitive level and slope that removed any measurement error associated with the individual tests and produced an index of overall cognitive ability and its ageing. Overall, this was a powerful way to assess cognitive decline, and we used a variety of PGSs from varied traits and disorders in an attempt to predict that decline’s variance. However, there are limitations to the study.

The self-selecting nature of the LBC1936 participants may have biased our results. That is, the participants were generally healthy, independently-living older adults and—by virtue of the fact they were able and interested to attend the initial testing—were healthier and more intellectually-engaged than the average person of their age. Non-random dropout, a problem for most longitudinal studies of ageing, compounds this issue: individuals who remained in the study across the four waves were healthier on average than those who dropped out. We thus probably missed individuals with the poorest health and, consequently, with the greatest degree of cognitive decline. This limitation—a restriction of range to the higher end of the health distribution—may have contributed to our lack of ability to predict cognitive decline in our sample.

There may also be additional complexities in the cognitive decline paths that we did not consider here, since we focused on the relatively simple linear average trajectories. For example, there may be nonlinear trajectories, or multiple latent trajectories (see e.g., ref. [72]) within the dataset that, if analysed in future, may reveal differential relations to the genetic predictors. Finally, in a few cases, the complexity of the structural equation models meant that convergence could not be achieved without fixing some of the paths that were intended to be freely-estimated (see Figure S2), and in some cases without extracting factor scores and using them in linear regression models instead of estimating all relations within latent-variable space (i.e., simultaneously to the estimation of the relevant structural equation model). We would not estimate that these issues would have changed our results to a great extent, but the latter practice (extracting factor scores) may have led to slight alterations to some of the standard errors we reported.

## Conclusions

A key goal of cognitive ageing research is to be able to predict who will experience steeper general cognitive decline. It is also of interest to build up a picture of which traits and disorders may share a genetic aetiology with variations in cognitive ageing. In this study of a high-quality, longitudinal dataset, we attempted to provide evidence on both of these questions using a panel of polygenic scores: however, despite substantial associations of several polygenic scores (those for education, schizophrenia, coronary artery disease, smoking, height, and BMI) with general cognitive level at baseline, and the relation of the education PGS to cognitive change between age 11 and age 70, none of the scores made useful, or even statistically significant, predictions of subsequent cognitive decline across the eighth decade of life in the preregistered analyses. Future, larger GWASs might furnish us with summary statistics to produce more predictive PGSs, and different analytic methods might increase the predictive value of those we already have. Given that it is possible to formulate so many PGSs from only DNA, the approach retains its potential as an efficient and protean source of predictors. For the time being, however, the findings suggest that researchers interested in genetic prediction of longitudinal variability in cognitive ageing will derive more value from established predictors, such as *APOE* e4, than newer methods such as polygenic scores.

## Supplementary information

Table S4

Supplementary Methods and Results
