# Supplementary material for: Polygenic predictors of age-related decline in cognitive ability
Source: Mol Psychiatry. 2019 Feb 13;25(10):2584–98. doi: 10.1038/s41380-019-0372-x (PMC7515838; doi:10.1038/s41380-019-0372-x)
Supplement: Supplementary file 2 — Supplementary Methods and Results [file 41380_2019_372_MOESM2_ESM.docx]

**Supplementary Materials for:**

**Polygenic predictors of age-related decline in cognitive ability**

S. J. Ritchie et al.

**Supplementary Method**

**Genome-wide association studies**

Table S1 provides a list of the genome-wide association studies (GWASs) where we obtained the summary statistics used to calculate the polygenic scores (PGSs) in the Lothian Birth Cohort 1936 sample. In addition to our use of summary statistics from pre-existing genome-wide association studies, we ran four additional GWAS studies in the UK Biobank dataset for the following phenotypes (with the UK Biobank variable identifier in parentheses): educational attainment (specifically the attainment of a college or university degree as a binary variable; derived from variable 6138.0.0), grip strength (measured in kg on a dynamometer; variable 47.0.0), lung function (forced expiratory volume in 1 second; FEV_1_; variable 3063.0.0) and smoking status (never, previous, or current smoker; variable 20116.0.0). Specific measurement details can be found at the UK Biobank website at the following URL: <http://biobank.ctsu.ox.ac.uk/crystal/label.cgi>. As noted in the main document, we updated the PGSs to take advantage of newer GWAS studies that appeared since we first ran our analysis; this meant that we no longer used the education data from UK Biobank since a new education GWAS had been published. We still describe it here for completeness, and the original results are shown in Table SXXX for comparison.

All the variables were corrected for age at the time of testing, sex, the genotype measurement batch (variable 22000), the genotyping array (either BiLEVE or Axiom, coded from variable 22000), the UK Biobank Assessment Centre (variable 54), along with 40 SNP principal components to adjust for potential population stratification. The correction was achieved by saving the residuals from a regression with the phenotype of interest as the dependent variable and the above covariates as the independent variables.

Each GWAS followed the procedure described in the Methods section of Luciano et al. (2018). Briefly, we used the imputed dataset (with over 92m variants referenced to the UK10K haplotype, 1000 Genomes Project phase 3, and Haplotype Reference Consortium panels) which was made available in UK Biobank. Quality control (see the Supplementary Note to ref^S2^) resulted in a total of 18,485,882 imputed SNPs and a maximum of 329,821 participants. GWAS was conducted using BGENIE^S1^. For each of the variables, residualized in the manner described above, we had the following sample sizes available: Educational attainment: *n* = 274,273; grip strength: *n* = 330,326; lung function: *n* = 302,605; smoking status: n = 330,918.

Manhattan plots, illustrating the individual SNP results for the latter three phenotypes, are provided below as Figures S1-S3. The plot for educational attainment (which, to repeat, was no longer used in the main analysis since it had been superseded by a further GWAS) can be found in the forthcoming ref.^S3^. Summary statistics from each GWAS were passed to the PRSice software for polygenic score calculation, as described in the Method section of the main document.

**Supplementary References**

S1. Bycroft C, Freeman C, Petkova D, Band G, Elliott LT, Sharp K, Motyer A, Vukcevic D, Delaneau O, O'Connell J, Cortes A, Welsh A, McVean G, Leslie S, Donnelly P, Marchini J. Genome-wide genetic data on ~500,000 UK Biobank participants. *bioRxiv.* 2017;166298. doi: 10.1101/166298

S2. Luciano M, Hagenaars SP, Davies G, Hill WD, Clarke TK, Shirali M, Harris SE, Marioni RE, Liewald DC, Fawns-Ritchie C, Adams MJ, Howard DM, Lewis CM, Gale CR, McIntosh AM, Deary IJ. Association analysis in over 329,000 individuals identifies 116 independent variants influencing neuroticism. *Nat Genet.* 2018;50:6-11.

S3. Hill WD et al. (forthcoming). Genetic analyses of socioeconomic status in UK Biobank.

S4. Diedenhofen B, Musch, J. cocor: A comprehensive solution for the statistical comparison of correlations. *PLOS ONE*. 2015;10:e0121945.

**Supplementary Tables**

*Table S1.* Studies relating the phenotype of each selected polygenic score to cognitive decline, and the relevant genome-wide association study (GWAS) from which the summary statistics to calculate the PGS were derived. Also provided is the discovery sample size for each GWAS.

| Polygenic Score | Authors and date; DOI | | GWAS sample size |
| --- | --- | --- | --- |
|  | Phenotypic linkage study | GWAS |  |
| Education | Bosma et al. (2003); 10.1080/10715769800300191 | Lee et al. (2018); 10.1038/s41588-018-0147-3 | 1,131,881 |
| Neuroticism | Hagger-Johnson et al. (2012); 10.1093/geronb/gbr151 | Luciano et al. (2018); 10.1038/s41588-017-0013-8 | 329,821 |
| Conscientiousness | Wilson et al. (2015); 10.1037/pag0000013 | Lo et al. (2017);  10.1038/ng.3736 | 76,551 |
| Alzheimer’s disease | Albert et al. (2011); 10.1016/j.jalz.2011.03.008 | Marioni et al. (2018); 10.1038/s41398-018-0150-6 | 25,580 cases; 48,466 controls |
| Schizophrenia | Reichenberg et al. (2005); 10.1001/archpsyc.62.12.1297 | Pardiñas et al. (2018); 10.1038/s41588-018-0059-2 | 11,260 cases; 24,542 controls |
| Major depressive disorder | Paterniti et al. (2002); 10.1192/bjp.181.5.406 | Wray et al. (2018); 10.1038/s41588-018-0090-3 | 135,458 cases; 344,901 controls |
| Coronary artery disease | Qiu & Fratiglioni (2015); 10.1038/nrcardio.2014.223 | Nikpay et al. (2015); 10.1038/ng.3396 | 60,801 cases; 123,504 controls |
| Stroke | Brainin et al. (2015); 10.1111/ene.12626 | Malik et al. (2018); 10.1038/s41588-018-0058-3 | 67,162 cases; 454,450 controls |
| Type 2 diabetes | Feinkohl et al. (2015); 10.1186/s13195-015-0130-5 | Scott et al. (2017); 10.2337/db16-1253 | 26,676 cases; 132,532 controls |
| Smoking | Anstey et al. (2007); 10.1093/aje/kwm116 | UK Biobank | 330,918 |
| Height | Case & Paxson (2008); 10.1257/aer.98.2.463 | Wood et al. (2014); 10.1038/ng.3097 | 253,288 |
| BMI | Gunstad et al. (2010); 10.1159/000297742 | Yengo et al. (2018); 10.1101/274654 | 681,275 |
| FEV_1_ | Richards et al. (2005); 10.1097/01.psy.0000170337.51848.68 | UK Biobank | 302,605 |
| Grip strength | Auyeung et al. (2011); 10.1007/s12603-011-0110-9 | UK Biobank | 330,326 |

*Note:* For the four GWAS that were performed in UK Biobank for the present analysis, details can be found in the Supplementary Method section above.

*Table S2.* Pearson correlations between polygenic scores in the Lothian Birth Cohort 1936.

| Polygenic Score | 1. | 2. | 3. | 4. | 5. | 6. | 7. | 8. | 9. | 10. | 11. | 12. | 13. |
| --- | --- | --- | --- | --- | --- | --- | --- | --- | --- | --- | --- | --- | --- |
| 1. Education | - |  |  |  |  |  |  |  |  |  |  |  |  |
| 2. Neuroticism | –.14^***^ | - |  |  |  |  |  |  |  |  |  |  |  |
| 3. Conscientiousness | –.01 | –.04 | - |  |  |  |  |  |  |  |  |  |  |
| 4. Alzheimer’s disease | –.04 | .01 | –.02 | - |  |  |  |  |  |  |  |  |  |
| 5. Schizophrenia | –.06 | .08^**^ | –.07^*^ | .04 | - |  |  |  |  |  |  |  |  |
| 6. Major depressive disorder | –.09^**^ | .23^***^ | –.10^**^ | .01 | .18^***^ | - |  |  |  |  |  |  |  |
| 7. Coronary artery disease | –.14^***^ | .004 | –.03 | .02 | .06 | .09^**^ | - |  |  |  |  |  |  |
| 8. Stroke | –.12^***^ | .03 | –.04 | –.03 | .07^*^ | .07^*^ | .06 | - |  |  |  |  |  |
| 9. Type 2 diabetes | –.09^**^ | –.02 | –.02 | .01 | .10^**^ | .04 | .11^***^ | .09^**^ | - |  |  |  |  |
| 10. Smoking | –.25^***^ | .06^*^ | –.01 | –.01 | .12^***^ | .14^***^ | .08^**^ | .08^*^ | .07^*^ | - |  |  |  |
| 11. Height | .08^**^ | –.04 | .02 | .01 | –.15^***^ | –.09^**^ | –.03 | –.10^**^ | –.14^***^ | –.04 | - |  |  |
| 12. BMI | –.26^***^ | –.09^**^ | –.05 | .01 | .01 | .07^*^ | .10^**^ | .09^**^ | .23^***^ | .18^***^ | –.10^**^ | - |  |
| 13. FEV_1_ | .13^***^ | .09^**^ | –.07^*^ | .00 | .13^***^ | .03 | –.01 | .05 | –.04 | –.13^***^ | –.07^*^ | –.14^***^ | - |
| 14. Grip strength | .03 | –.12^***^ | .05 | –.07^*^ | .03 | –.07^*^ | .03 | .03 | –.02 | –.03 | –.04 | .04 | .15^***^ |

*Note:* *n* = 1,005 for all correlations. ^*^*p* < .05, ^**^*p* < .01, ^***^*p* < .001.

*Table S3.* Descriptive statistics for each cognitive test at each wave of the study.

| Test | Wave 1  (70 years) | | Wave 2  (73 years) | | Wave 3  (76 years) | | Wave 4  (79 years) | |
| --- | --- | --- | --- | --- | --- | --- | --- | --- |
|  | Mean (SD) | N | Mean (SD) | N | Mean (SD) | N | Mean (SD) | N |
| Matrix reasoning | 13.49 (5.13) | 1086 | 13.17 (4.96) | 863 | 13.04 (4.91) | 689 | 12.90 (5.03) | 535 |
| Block design | 33.79 (10.32) | 1085 | 33.64 (10.08) | 864 | 32.18 (9.95) | 691 | 31.20 (9.63) | 535 |
| Spatial span | 7.36 (1.42) | 1084 | 7.35 (1.38) | 861 | 7.31 (1.36) | 690 | 7.07 (1.36) | 536 |
| Logical memory | 71.46 (17.96) | 1087 | 74.30 (17.88) | 864 | 74.58 (19.20) | 688 | 72.71 (20.39) | 542 |
| Verbal paired associates | 26.44 (9.13) | 1050 | 27.18 (9.46) | 843 | 26.41 (9.56) | 663 | 27.14 (9.55) | 497 |
| Digit span backwards | 7.73 (2.26) | 1090 | 7.81 (2.29) | 866 | 7.77 (2.37) | 695 | 7.56 (2.18) | 548 |
| NART | 34.48 (8.15) | 1089 | 34.38 (8.18) | 864 | 35.02 (8.03) | 695 | 35.59 (8.19) | 546 |
| WTAR | 41.02 (7.17) | 1089 | 41.01 (6.97) | 864 | 41.09 (7.02) | 694 | 41.63 (7.03) | 546 |
| Verbal fluency | 42.42 (12.54) | 1087 | 43.18 (12.94) | 865 | 42.90 (12.76) | 696 | 43.61 (13.33) | 547 |
| Digit-symbol substitution | 56.60 (12.93) | 1086 | 56.40 (12.31) | 862 | 53.81 (12.93) | 685 | 49.70 (38.06) | 535 |
| Symbol search | 24.71 (6.39) | 1086 | 24.61 (6.18) | 862 | 24.60 (6.46) | 687 | 19.25 (56.65) | 529 |
| Inspection time | 112.14 (11.00) | 1041 | 111.22 (11.79) | 838 | 110.14 (12.55) | 654 | 105.06 (43.14) | 465 |
| Choice reaction time (ms) | 64.21 (0.09) | 1084 | 64.93 (8.98) | 865 | 67.88 (10.28) | 685 | 70.60 (11.36) | 543 |

*Table S4.* Correlation matrix for all cognitive test scores.

See attached Excel Spreadsheet: “TableS4_cormat.xlsx”, available at this study’s associated Open Science Framework page <https://osf.io/hquy9/>. The first sheet, cormat_r, provides the correlations (Pearson’s *r*), and the second sheet, cormat_n, provides the effective sample size for each correlation (the diagonal has the total *n* of each variable at wave 1, and corresponds to Table S2).

*Key to variable names:*

matreas = Matrix Reasoning

blkdes = Block Design

span = Spatial Span

lm = Logical Memory

vpa = Verbal Paired Associates

digback = Digit Span Backwards

nart = National Adult Reading Test

wtar = Wechsler Test of Adult Reading

vf = Verbal Fluency

digsym = Digit-Symbol Substitution

symsear = Symbol Search

it = Inspection Time

crt = Choice Reaction Time

*Note:* the variable suffix “_w1” refers to wave 1, and so on for the four waves.

*Table S5.* Associations of polygenic scores that were significant in the main analysis with general cognitive outcomes, corrected for *APOE* e4 status.

| Polygenic score | Cognitive outcome | Polygenic score association | | | *APOE* e4 association | | |
| --- | --- | --- | --- | --- | --- | --- | --- |
|  |  | Std. *β* | SE | *p*-value | Std. *β* | SE | *p*-value |
| Education | Level | .269 | .031 | 1.35×10^-17^ | –.171 | .068 | .012 |
| Schizophrenia | Level | –.139 | .035 | 9.86×10^-05^ | –.132 | .070 | .056 |
|  | Slope | –.090 | .036 | .013 | –.328 | .071 | 4.31×10^-06^ |
| Coronary artery disease | Level | –.092 | .032 | .004 | –.125 | .070 | .073 |
| Type 2 diabetes | Level | –.074 | .033 | .024 | –.132 | .070 | .060 |
| Smoking | Level | –.163 | .033 | 6.77×10^-07^ | –.143 | .069 | .039 |
| Height | Level | .084 | .034 | .016 | –.126 | .070 | .071 |
| BMI | Level | –.117 | .033 | 5.62×10^-04^ | –.117 | .070 | .094 |

Note: All of the models in this table were run in a regression using cognitive factor scores extracted from the structural equation model shown in Figure 1. All variables corrected for age and sex, and all models also included four multidimensional scaling components.

*Table S6.* Results from a simultaneous model of all polygenic scores predicting general cognitive level and general cognitive slope.

| Polygenic score | General cognitive level | | | General cognitive slope | | |
| --- | --- | --- | --- | --- | --- | --- |
|  | Std. *β* | SE | *p*-value | Std. *β* | SE | *p*-value |
| Education | **.217** | **.032** | **7.44×10^-11^** | .025 | .035 | .466 |
| Neuroticism | .001 | .035 | .979 | .008 | .038 | .825 |
| Conscientiousness | –.017 | .031 | .582 | .008 | .033 | .803 |
| Alzheimer’s disease | –.003 | .030 | .921 | –.047 | .033 | .149 |
| Schizophrenia | **–.109** | **.034** | **.001** | –.089 | .037 | .016 |
| Major depressive disorder | .023 | .032 | .483 | –.010 | .034 | .763 |
| Coronary artery disease | –.051 | .031 | .097 | –.014 | .033 | .680 |
| Stroke | –.007 | .032 | .830 | –.012 | .033 | .714 |
| Type 2 diabetes | –.035 | .034 | .273 | –.010 | .034 | .775 |
| Smoking | **–.090** | **.033** | **.006** | –.030 | .035 | .390 |
| Height | .042 | .034 | .212 | –.007 | .036 | .857 |
| BMI | –.036 | .034 | .282 | –.022 | .036 | .539 |
| FEV_1_ | .038 | .033 | .254 | .040 | .035 | .256 |
| Grip strength | –.052 | .031 | .100 | .007 | .033 | .843 |

*Note:* Rows in bold are effects that survived false discovery rate correction for multiple testing. All variables corrected for age and sex, and all models also included four multidimensional scaling components.

*Table S7*. Results from the mediation model where education mediates the path between the education-linked polygenic score and cognitive change across the lifespan (see Figure S7).

| Path | Std. *β* | SE | *p*-value |
| --- | --- | --- | --- |
| Education PGS -> Years of Education (a) | .273 | .030 | 2.61**×**10^-20^ |
| Years of Education -> g age 70 (b) | .271 | .029 | 5.45**×**10^-21^ |
| Education PGS -> g age 70 (c) | .068 | .026 | .018 |
| Age 11 IQ -> g age 70 | .744 | .021 | 4.07**×**10^-284^ |
| Indirect path (a*b) | .019 | .007 | .008 |
| Total paths (a*b + c) | .291 | .030 | 7.54**×**10^-22^ |
| Proportion of mediation (Indirect/Total) | .064 | .023 | .018 |

*Table S8.* Associations between each polygenic score the baseline level and slope of general cognitive ability, with polygenic scores calculated at the *p* < .01 threshold.

| Polygenic score | Association with baseline *g* | | | | Association with *g* slope | | | |
| --- | --- | --- | --- | --- | --- | --- | --- | --- |
|  | Std. *β* | SE | *p*-value | Δ | Std. *β* | SE | *p*-value | Δ |
| Education | **.291** | **.033** | **3.69×10^-19^** | –.011 | .022 | .044 | .623 | –.016 |
| Neuroticism | –.002 | .032 | .474 | –.030 | .011 | .032 | .728 | –.015 |
| Conscientiousness | –.035 | .035 | .317 | –.045 | –.071 | .044 | .107 | –.086 |
| Alzheimer’s disease | –.056 | .035 | .110 | –.039 | **–.109** | **.043** | **.012** | –.036 |
| Schizophrenia | **–.135** | **.045** | **.002** | +.001 | –.073 | .055 | .185 | +.037 |
| Major depressive disorder | –.044 | .036 | .223 | –.007 | .037 | .045 | .813 | +.045 |
| Coronary artery disease | .031 | .035 | .377 | +.139 | –.059 | .044 | .176 | –.048 |
| Stroke | –.019 | .035 | .580 | +.040 | –.043 | .044 | .322 | –.027 |
| Type 2 diabetes | –.108 | .035 | .002 | –.019 | .012 | .043 | .282 | +.028 |
| Smoking | **–.150** | **.036** | **3.35×10^-05^** | –.028 | –.037 | .046 | .425 | –.002 |
| Height | .079 | .036 | .029 | +.022 | –.012 | .046 | .792 | –.001 |
| BMI | **–.139** | **.037** | **1.78×10^-04^** | –.004 | .057 | .047 | .226 | +.029 |
| FEV_1_ | .020 | .041 | .633 | +.054 | .001 | .052 | .988 | –.050 |
| Grip strength | .013 | .035 | .722 | +.055 | ~.000 | .044 | .995 | –.035 |

*Note.* Δ refers to the difference in the standardized *β* of the association between the *p* = 1.00 threshold score and the score calculated at this threshold (*p* = .01).

*Table S9.* Comparisons between genotypic (polygenic risk score) predictors and phenotypic predictors in their associations with baseline general intelligence (*g*) at age 70 years and change in general intelligence from age 70 to 79 years.

| Predictor | Phenotypic variable | Association with baseline *g* | | | | | | | Association with *g* slope | | | | | | |
| --- | --- | --- | --- | --- | --- | --- | --- | --- | --- | --- | --- | --- | --- | --- | --- |
|  |  | Genotype | | | Phenotype | | | Comparison | Genotype | | | Phenotype | | | Comparison |
|  |  | Std. *β* | SE | *p*-value | Std. *β* | SE | *p*-value | *p*-value | Std. *β* | SE | *p*-value | Std. *β* | SE | *p*-value | *p*-value |
| Education | Years of education | **.302** | **.032** | **1.31×10^-20^** | **.537** | **.027** | **1.11×10^-88^** | **3.08×10^-13^** | .006 | .044 | .888 | –.045 | .043 | .291 | .178 |
| Neuroticism | NEO-PI-R Neuroticism | –.077 | .039 | .047 | –**.206** | **.036** | **1.11×10^-08^** | **.0017** | –.004 | .048 | .929 | –.041 | .045 | .364 | .377 |
| Conscientiousness | NEO-PI-R Conscientiousness | –.017 | .035 | .634 | –.017 | .038 | .649 | 1.00 | .015 | .044 | .726 | .099 | .044 | .025 | .049 |
| Alzheimer’s disease | MMSE | –.017 | .035 | .634 | **.551** | **.025** | **1.65×10^-110^** | **1.68×10^-46^** | –.073 | .044 | .094 | **.146** | **.044** | **.001** | **1.37×10^-06^** |
| Major depressive disorder | HADS depression score | –.037 | .035 | .290 | –.039 | .035 | .259 | .964 | –.008 | .044 | .856 | –.007 | .043 | .872 | .982 |
| Coronary artery disease | Cardiovascular disease | **–.108** | **.035** | **.002** | –**.110** | **.035** | **.001** | .961 | –.011 | .044 | .808 | –.007 | .044 | .872 | .924 |
| Stroke | Stroke | –.056 | .035 | .109 | –.057 | .035 | .103 | .981 | –.016 | .043 | .719 | –.011 | .043 | .790 | .905 |
| Type 2 diabetes | Type 2 diabetes | **–.089** | **.035** | **.012** | –**.089** | **.035** | **.010** | 1.00 | –.010 | .045 | .826 | –.008 | .044 | .853 | .961 |
| Smoking | Smoking | **–.178** | **.035** | **3.66×10^-07^** | –**.176** | **.034** | **2.09×10^-07^** | .966 | –.035 | .045 | .438 | –.020 | .043 | .654 | .753 |
| Height | Height | **.093** | **.035** | **.008** | **.093** | **.035** | **.007** | 1.00 | .009 | .045 | .833 | .004 | .044 | .937 | .890 |
| BMI | BMI | **–.135** | **.036** | **1.76×10^-07^** | –**.130** | **.035** | **1.67×10^-04^** | .890 | .028 | .045 | .540 | .038 | .044 | .387 | .784 |
| FEV_1_ | FEV_1_ | .074 | .037 | .048 | .053 | .035 | .127 | .600 | .051 | .047 | .277 | .042 | .043 | .337 | .822 |
| Grip strength | Grip strength | –.041 | .036 | .255 | –.046 | .035 | .184 | .044 | .035 | .044 | .425 | .033 | .043 | .447 | .963 |

*Note:* Genotypic values are reproduced from Table 3 in the main document. Values in bold survived False Discovery Rate correction of their *p*-values. Schizophrenia is not included because the phenotype we used to test it (see Table 3), Block Design, is included in the cognitive model. For the comparison between the genotypic and phenotypic predictions, we used Williams’s test (using the *cocor* package for *R*^S4^), treating the standardized *β*-values as correlation coefficients and taking into account the dependency between the genotypic and phenotypic predictors.

*Table S10.* Results from Cox proportional hazard models predicting time to mortality from each polygenic score (or *APOE* e4 status).

| Genetic variable | Hazard ratio | SE | *p*-value |
| --- | --- | --- | --- |
| *APOE* e4 | 1.003 | .141 | .981 |
| Education | 0.941 | .064 | .340 |
| Neuroticism | 1.008 | .070 | .147 |
| Conscientiousness | 0.941 | .064 | .338 |
| Alzheimer’s disease | 0.909 | .063 | .134 |
| Schizophrenia | 1.095 | .071 | .203 |
| Major depressive disorder | 1.006 | .064 | .930 |
| Coronary artery disease | 1.058 | .064 | .380 |
| Stroke | 1.024 | .064 | .709 |
| Type 2 diabetes | 0.999 | .065 | .988 |
| Smoking | 1.083 | .066 | .221 |
| Height | 0.928 | .071 | .295 |
| BMI | 1.174 | .068 | .018 |
| FEV_1_ | 0.946 | .069 | .416 |
| Grip strength | 0.951 | .65 | .433 |

*Note:* All models also adjusted for sex and for four multidimensional scaling components (to adjust for population stratification).

*Table S11*. Estimates from the previous iterations of specific polygenic scores (that is, calculated on the basis of previous, smaller GWAS studies), updated after the initial analysis.

| Genetic variable | Association with baseline *g* | | | Association with *g* slope | | |
| --- | --- | --- | --- | --- | --- | --- |
|  | Std. *β* | SE | *p*-value | Std. *β* | SE | *p*-value |
| Education | .264 | .033 | 1.54×10^-15^ | .031 | .043 | .474 |
| Alzheimer’s disease | –.035 | .035 | .316 | .002 | .045 | .957 |
| Major depressive disorder | –.034 | .035 | .342 | –.049 | .044 | .267 |
| Stroke | .026 | .036 | .463 | .046 | .044 | .295 |
| Type 2 diabetes | –.043 | .032 | .171 | –.019 | .032 | .550 |
| Height | .101 | .038 | .008 | –.011 | .049 | .826 |
| BMI | –.109 | .035 | .002 | –.014 | .044 | .746 |

*Note:* The references/DOIs for the previous GWASs are the following. Education: in-house UK Biobank study as described above; Alzheimer’s disease: 10.1038/ng.2802; Major depressive disorder: 10.1038/mp.2012.21; Stroke: 10.1016/S1474-4422(12)70234-X; Type 2 diabetes: 10.1038/ng.2383; BMI: 10.1038/nature14177. The GWAS studies used in the main analysis are referenced in Table S1.

**Supplementary Figures**

*Figure S1.* Genome-wide association Manhattan plot for grip strength in UK Biobank.

*
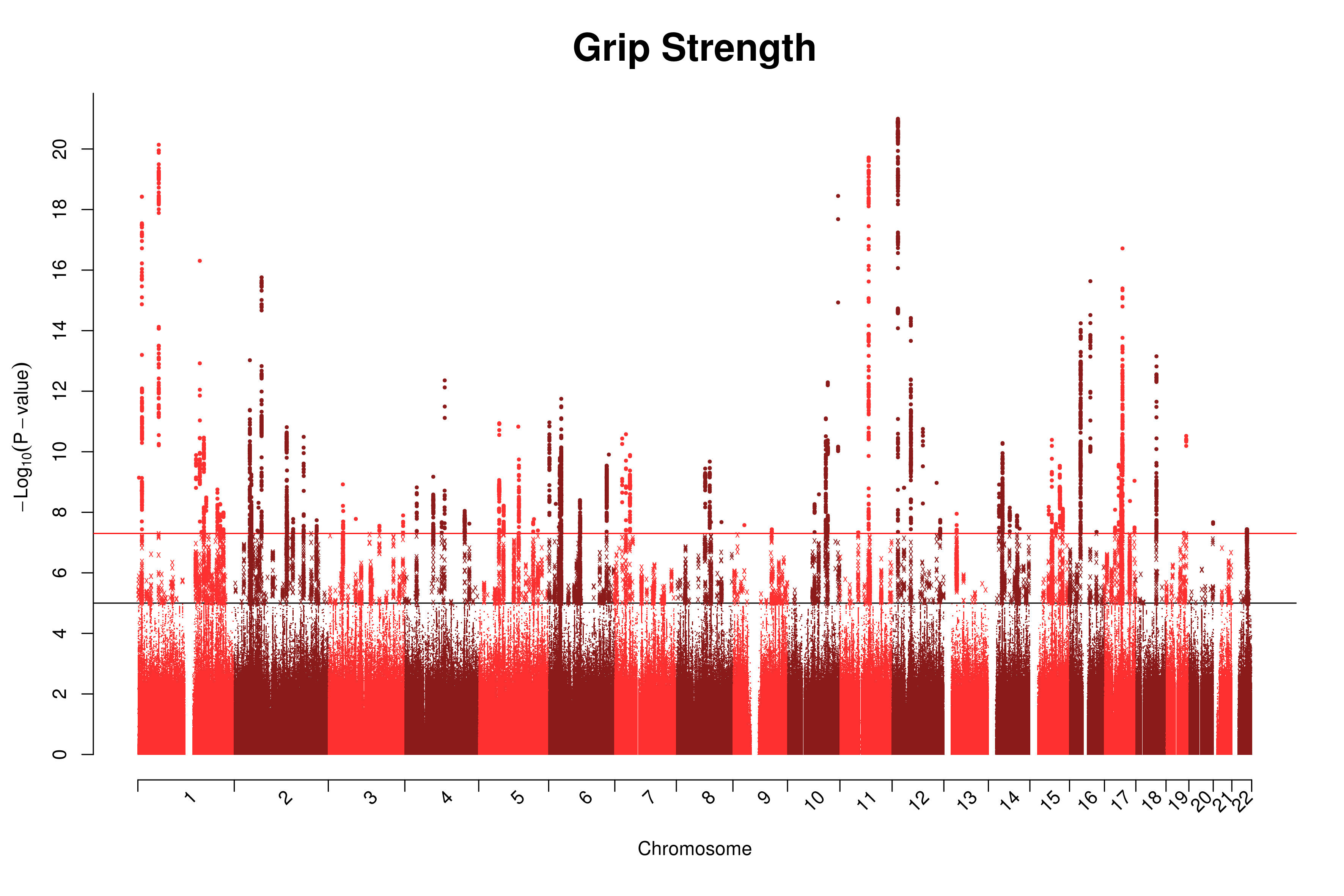
*

*Figure S2.* Genome-wide association Manhattan plot for lung function (forced expiratory volume in 1 second; FEV_1_) in UK Biobank.

*
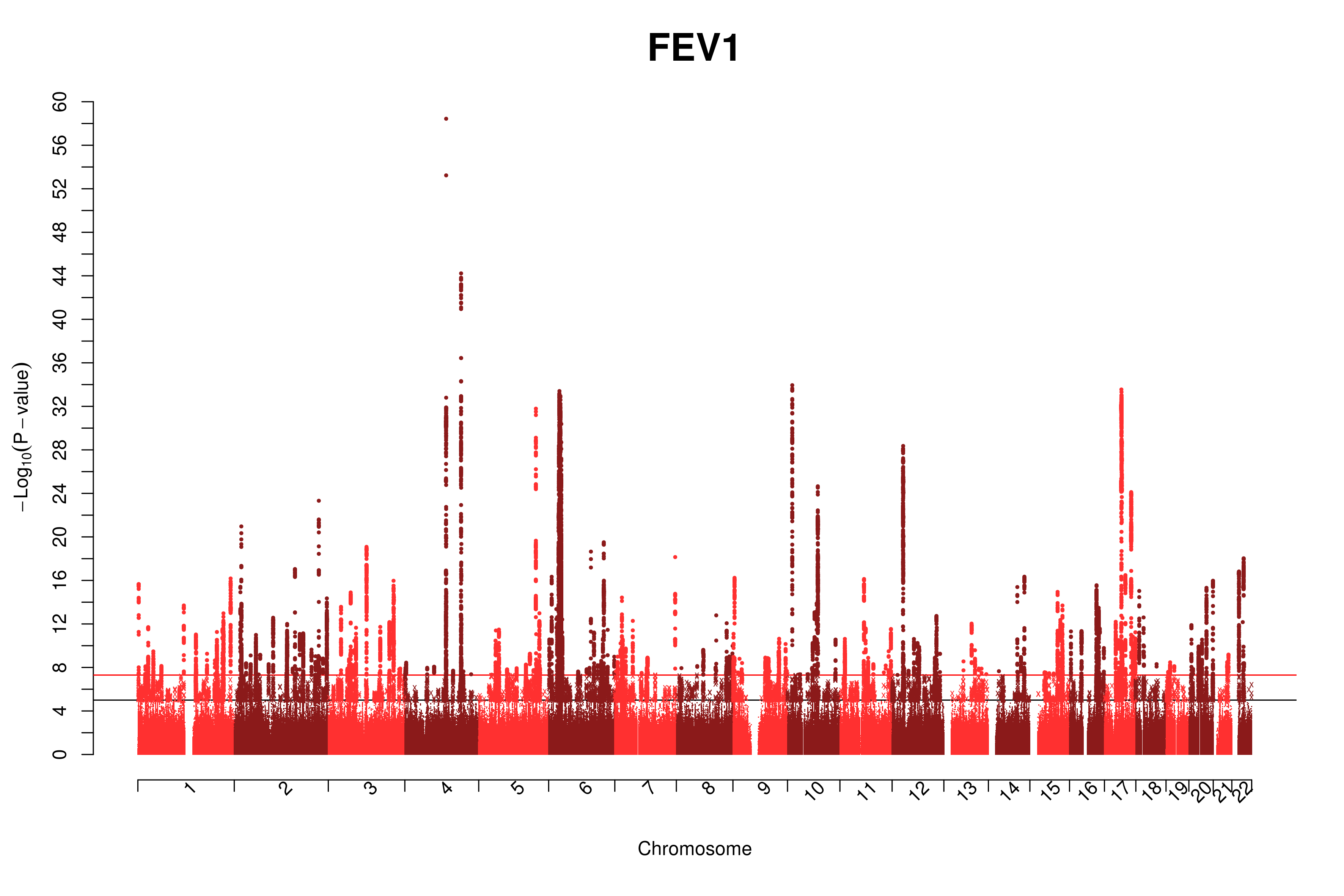
*

*Figure S3.* Genome-wide association Manhattan plot for smoking status in UK Biobank.

*
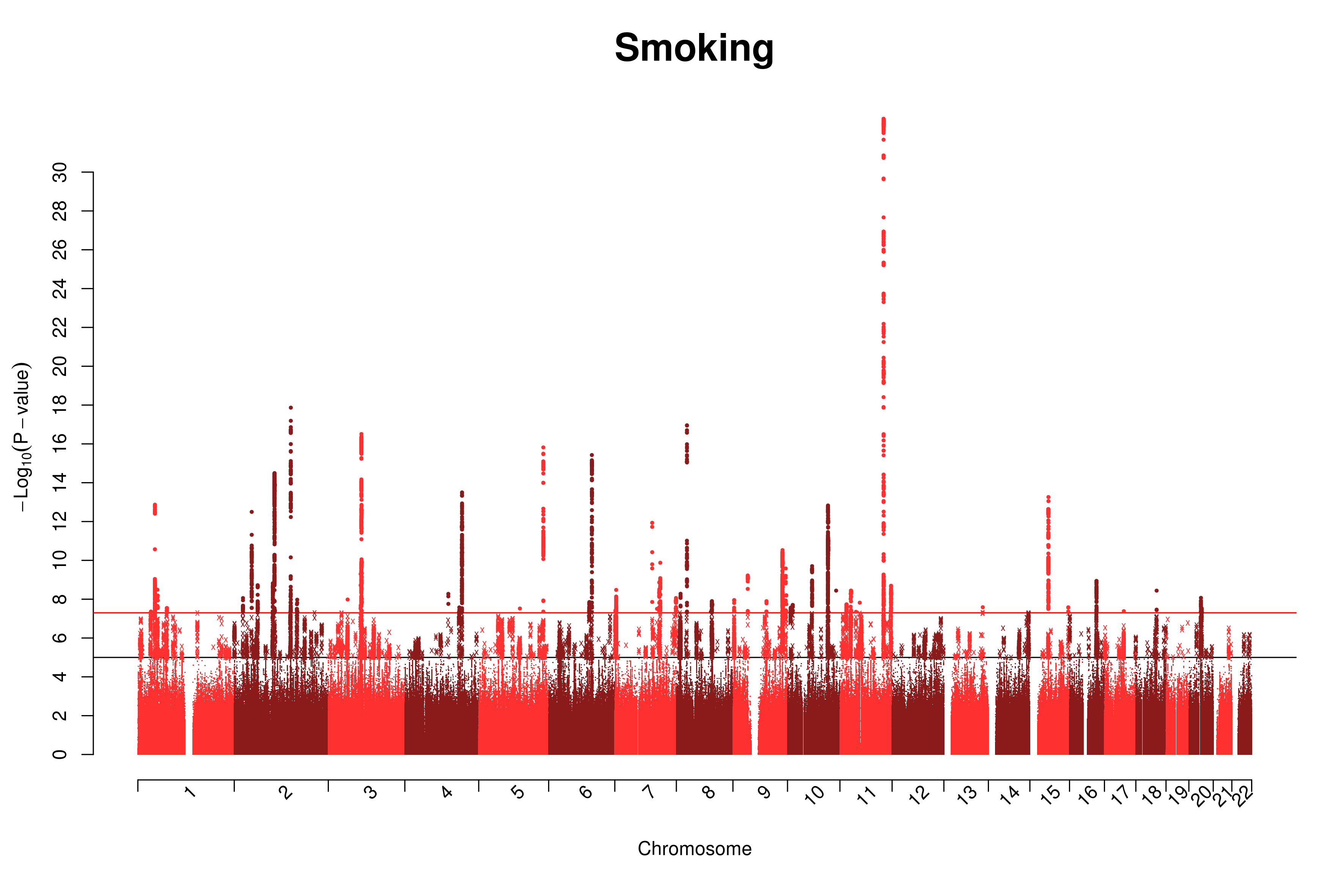
*

*Figure S4.* Ageing trajectories of standardized scores on the raw data from each cognitive test, with intercept (at the youngest age) set to zero, showing all data points. The black line denotes the mean change from the original point across the four waves of data collection.


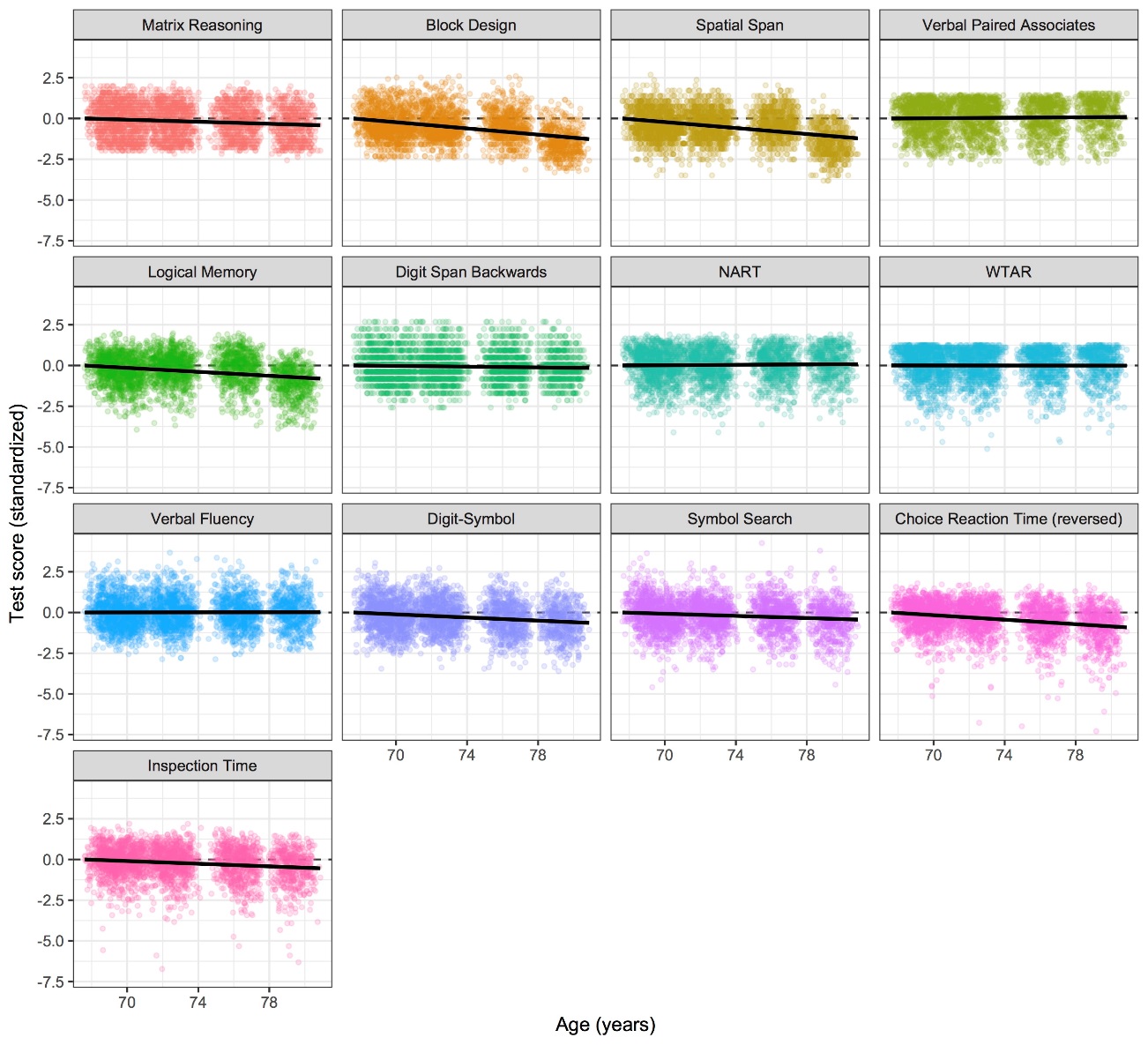


*Figure S5.* Standardized factor loadings for the structural model of the baseline “factors of curves” model. All tests were corrected for sex and for age in days at the testing wave, but no other covariates or predictors were included. Note that the paths estimated as 1 were fixed to this value due to the model estimating negative (but near-zero) residual variances for these parameters; the loadings were thus fixed to 1 to allow the model to converge on estimates that were within standardized bounds.


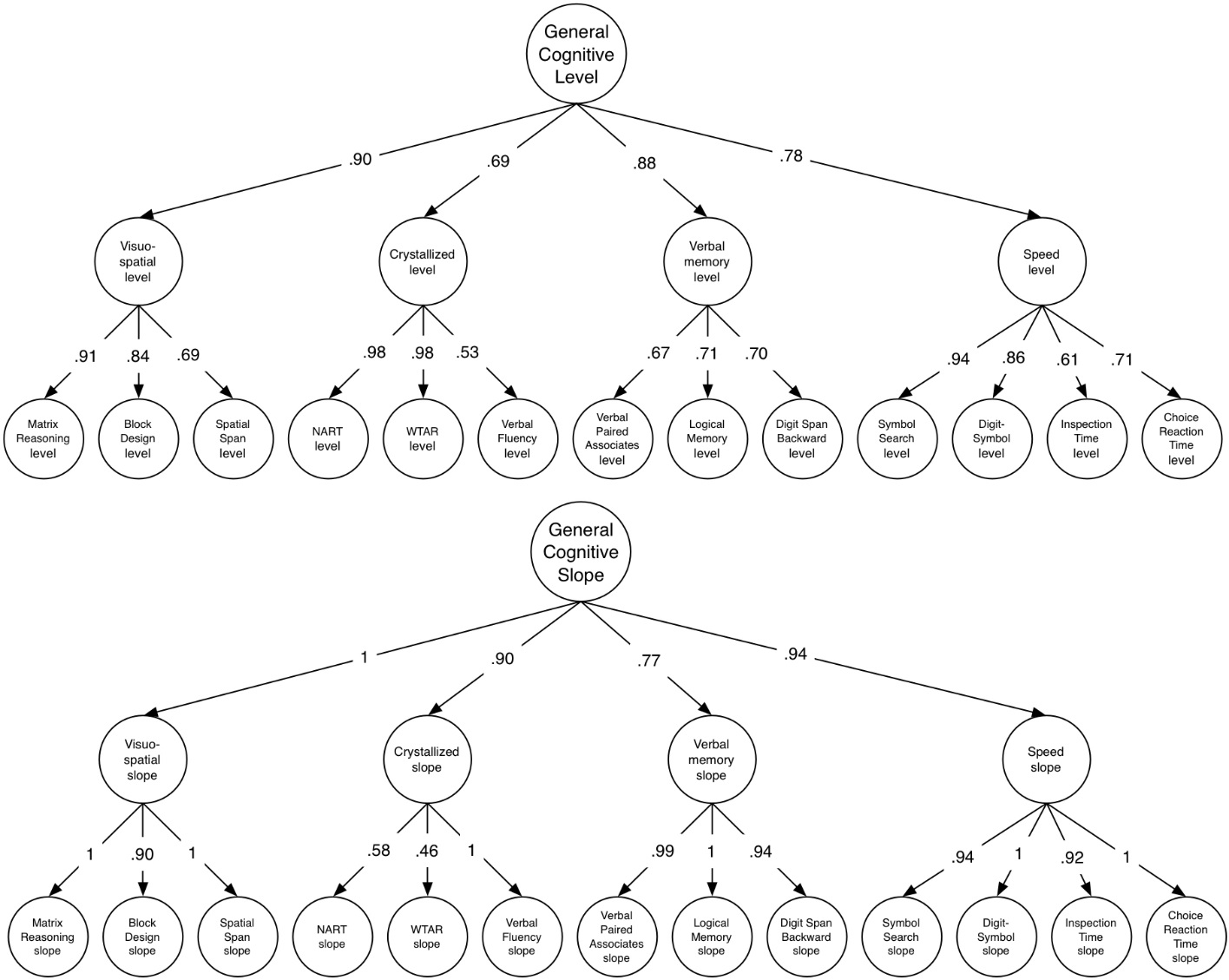


*Figure S6*. Standardized factor loadings for the age-70 model used to estimate lifetime cognitive change. No covariates were included in the model shown in this figure.


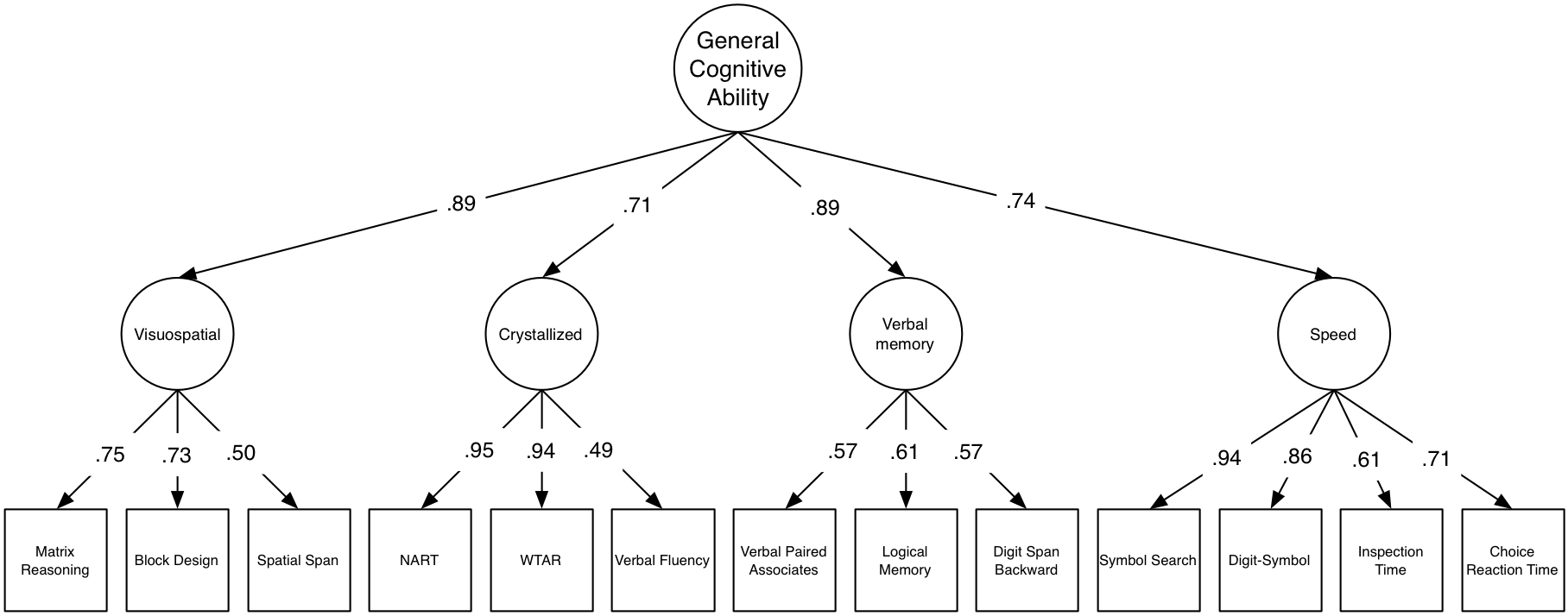


*Figure S7*. Mediation model where education mediates the path between the education-linked polygenic score and cognitive change across the lifespan. Note that *g* (general cognitive ability) age 70 is a latent variable indicated using the same higher-order model shown in Figure S6, but for illustrative purposes the cognitive tests and their structure are not shown here. Paths a and b represent the indirect path between the education polygenic score (PGS) and *g* age 70; path c is the direct path.
